# Synthesis of Ti6Al4V/SrFHA Composites by Microwave-Assisted Liquid Phase Deposition and Calcination

**DOI:** 10.3390/ma15186206

**Published:** 2022-09-07

**Authors:** Guangyan Zhu, Qian Peng, Ting Luo, Hao Pan, Yuehong Wang, Zhiwei Peng

**Affiliations:** 1 School of Minerals Processing and Bioengineering, Central South University, Changsha 410083, China; 2 Xiangya Stomatological Hospital, Central South University, Changsha 410008, China; 3 Xiangya School of Stomatology, Central South University, Changsha 410008, China; 4 Hunan Key Laboratory of Oral Health Research, Central South University, Changsha 410008, China

**Keywords:** microwave processing, liquid phase deposition, co-doping, HA, Ti6Al4V

## Abstract

The feasibility of synthesis of Ti6Al4V/SrFHA (Ca_9.37_Sr_0.63_(PO_4_)_6_F_2_) composites via coating strontium and fluorine co-doped HA to Ti6Al4V substrate by microwave-assisted liquid phase deposition and calcination was evaluated, with a focus on the effect of the deposition temperature from 30 °C to 70 °C. The outcomes demonstrate that strontium and fluorine can be successfully doped into HA to form a SrFHA coating with modified micromorphology which is deposited on the alloy. When the deposition temperature was 50 °C, the coating with the largest uniform continuous SrFHA coverage was obtained. After calcination, the adhesion strength and Vickers microhardness of the Ti6Al4V/SrFHA composite increased from 0.68 MPa and 323 HV to 2.41 MPa and 329 HV, respectively, with a decrease in the water contact angle from 10.88° to 7.24°, exhibiting enhancement of both mechanical properties and wettability. Moreover, the composite obtained at the deposition temperature of 50 °C exhibited good bioactivity based on the simulate body fluid (SBF) test. On account of the above features primarily as a result of the combined effect of the co-doping of strontium and fluorine, high crystallinity of SrFHA, large surface roughness, and formation of the titanium oxide transition layer, the Ti6Al4V/SrFHA composite shows great potential in dental implantology.

## 1. Introduction

Titanium and its alloys, such as Ti6Al4V, have been widely used in clinical practice as dental implants for repairing common dental diseases such as dentition defects and deletions because of their superior mechanical properties and good biocompatibility [1,2,3]. Compared with pure Ti, Ti6Al4V has higher strength, greater corrosion resistance, lower specific gravity, and smaller elastic modulus which is, however, still higher than that of human bone (17–28 GPa), resulting in stress shielding that degrades the implant stability [4,5,6]. It also suffers from no biological activity, the same as Ti. For this reason, after implantation, there is only a mechanical interlock with human bone tissue, which easily causes implantation failure [7,8]. Hydroxyapatite (HA, Ca_10_(PO_4_)_6_(OH)_2_), as the main natural component of human hard tissue, is widely used as a bioactive coating because of its excellent bioactivity. Currently, it is one of the most common strategies to modify the surface of Ti6Al4V using a HA coating to ensure sufficient bioactivity and mechanical properties of the resulting composite [9].

There are many methods for coating HA on Ti and its alloys, including the liquid phase deposition method [10,11,12], sol–gel method [13,14,15,16], electrochemical method [17,18], plasma spraying method [19,20,21], and selective laser melting method [22]. Among them, the liquid phase deposition method is featured by simple procedure and high versatility in coating. It can directly coat HA on the substrate of different shapes and thicknesses although it demands a relatively long deposition time and has low adhesion strength between the coating and Ti6Al4V which restrict its applications. To shorten synthesis time and improve the quality of the product, introducing external fields, such as microwaves, for preparation of HA on the titanium alloy as a more bioactive coating has attracted increasing attention in recent years [23,24,25,26,27]. In fact, many studies have demonstrated that the use of microwaves in the liquid phase deposition makes full use of the microwave volumetric and selective thermal effect and potential non-thermal effect to facilitate the coating process [24]. Moreover, for further improving the product quality, ion-doped HA is preferred for dental applications compared with pure HA. For instance, Sr and F are native trace elements in teeth. Sr doping can enhance the proliferation of osteoprogenitor cells and bone formation as well as apoptosis of mature osteoclasts after implantation, while adding F may improve the thermal stability and antibacterial properties of HA and promote cell adhesion on the implant material [28,29,30,31]. 

The aim of the present study was to coat Sr and F co-doped HA (SrFHA) on Ti6Al4V by microwave-assisted liquid phase deposition and calcination to synthesize the Ti6Al4V/SrFHA composites as novel dental implant materials to combine the inspiring advantages of Ti6Al4V and SrFHA in dental applications. The effects of the deposition temperature on the preparation process and properties of the composites were studied in detail.

## 2. Experimental

### 2.1. Preparation

**Figure 1:**
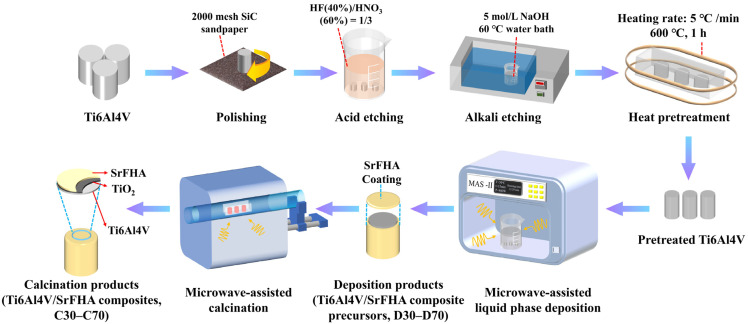
Schematic illustration of the synthesis process of Ti6Al4V/SrFHA composites.

For preparing the Ti6Al4V/SrFHA composites, all chemical reagents were of analytical grade and purchased from Aladdin Company (China). At first, cylindrical Ti6Al4V bars of 10 mm in diameter and 10 mm in height were selected for use as the substrates for coating of SrFHA by microwave-assisted liquid phase deposition. Before coating, the cylindrical bars were polished with #600, #1000, #1500, and #2000 silicon carbide (SiC) sandpaper and then ultrasonically cleaned in acetone, ethanol, and deionized water for 15 min in sequence. They were subsequently etched for 10 min in a mixed acid constituted by 30 mL of HNO_3_ (60%) and 10 mL of HF (40%). After heating in a water bath of 50 mL of 5 mol/L NaOH solution at 60 °C for 6 h and ultrasonic cleaning with deionized water for 15 min, the etched cylindrical bars were transferred to a muffle furnace for heat pretreatment at 600 °C for 1 h and cooled at room temperature as the substrates (called pretreated Ti6Al4V) for preparation of Ti6Al4V/SrFHA composites, as shown in Figure 1. The pretreatment was carried out to improve the surface roughness of titanium alloy substrate that could facilitate the deposition of coating [32]. For the deposition experiments, the deposition solution was firstly prepared according to the composition of 10 times that of the simulated body fluid (SBF) by adding 0.08 mol/L CaCl_2_, 0.02 mol/L SrCl_2_, 0.06 mol/L NaH_2_PO_4_, 0.02 mol/L NH_4_F, and 0.18 mol/L NaHCO_3_ to the standard SBF, which has been widely used in similar studies [33]. The pretreated Ti6Al4V substrates were then put into the solution for deposition at different temperatures (30 °C, 40 °C, 50 °C, 60 °C, and 70 °C) in a microwave reactor (MAS-Ⅱ, Hunan Huae Change Microwave Technology Co., Ltd., Changsha, China) for 5 min. The deposition products at the corresponding temperatures were recorded as D30, D40, D50, D60, and D70, which were then placed in a microwave tube furnace (model: CY-SVT1200C-SD, Hunan Huae Change Microwave Technology Co., Ltd., Changsha, China) for calcination at 800 °C for 20 min [34]. After cooling, the corresponding calcination products were collected as the Ti6Al4V/SrFHA composites and recorded as C30, C40, C50, C60, and C70, respectively, for characterization of properties, including adhesion strength, Vickers microhardness, wettability, and in vitro apatite-forming capability. The experiments were repeated three times and the average results were recorded.

### 2.2. Characterization

The phase compositions and crystallinities of the deposition and calcination products (D30–D70 and C30–C70) were analyzed using an X-ray diffractometer (XRD, Empyrean, PANalytical, The Netherlands). The lattice parameters (*a*, *b*, and *c*) of SrFHA were calculated based on the reflection planes (121) and (300) in the XRD patterns using the standard HCP unit cell plane spacing relationship [35]:

(1) $$\frac{1}{d^{2}}=\frac{4(h^{2}+hk+k^{2})}{a}+\frac{l^{2}}{c^{2}}$$

where *d* is the lattice spacing, and *h*, *k* and *l* are the Miller indices of the reflection planes. The lattice strain (*E*) was calculated using the following equation [36]:

(2) $$E=\frac{\beta_{\left(121\right)}}{4\times \tan\theta }$$

where *β* is the full width at half maximum (FWHM) intensity in radians and *θ* is the diffraction peak angle. The microstructures, morphological features, and elemental analyses of the products were determined using a scanning electron microscope (SEM, TESCAN, MIRA4 LMH, Brno, Czech Republic) coupled with energy dispersive X-ray spectroscopy (EDS, Ultim Max 40, Oxford Instruments, Abingdon, UK). The surface roughness of the products was measured using an atomic force microscope (AFM, Dimension Icon, Bruker, Karlsruhe, Germany). After binarization of the related SEM images, the coating coverage was calculated using the software ImageJ 1.49 (National Institutes of Health, Bethesda, MD, USA) [37]. The adhesion strength of the products was determined according to the method reported in the literature [34,38]. Specifically, it was measured using a universal testing machine after fixing one end of the composite with a Ti6Al4V rod of 6 mm in diameter and 10 mm in length using epoxy resin. The tested pieces were drawn at a crosshead speed of 1 mm/min until the coating detached. The Vickers microhardness of the products was evaluated using a hardness tester (Qness 30, QATM, Mammelzen, Germany) with a pyramid-shaped diamond indenter under the conditions of load of 1 kg and dwell time of 15 s. The wettability, represented by water contact angles, of the products was measured using a drop shape analyzer (DSA-30, CRUSS, Hamburg, Germany). The biological activities, revealed mainly by the apatite-forming capabilities in this study, were determined by the in vitro experiments in standard SBF obtained via dissolving reagent chemicals of NaCl, NaHCO_3_, KCl, K_2_HPO_4_·3H_2_O, MgCl_2_·6H_2_O, CaCl_2_, and Na_2_SO_4_ in deionized water. The experiments were carried out in a water bath maintained at a temperature of 37 ± 0.5 °C and the SBF was refreshed every 48 h in the total immersion period (soaking time) of 14 days. The concentrations of calcium, phosphorus, and strontium in the SBF were measured using an inductively coupled plasma optical emission spectrometer (ICP-OES, PerkinElmer, Avio 200, Waltham, MA, USA). The change in pH was determined using a pH meter (PHS-2F, Oustor, Shanghai, China). After the products were removed from the solution and gently rinsed with distilled water, the morphologies and elemental compositions of dried products were analyzed using SEM to show their ability to induce the formation of HA. 

## 3. Results and Discussion

### 3.1. Phase Compositions and Microstructures of Ti6Al4V/SrFHA Composites 

**Figure 2:**
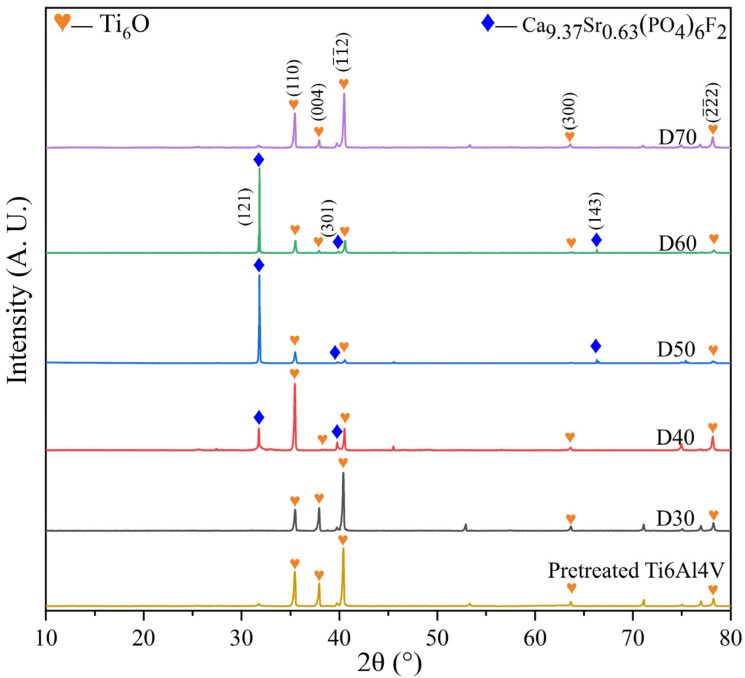
XRD patterns of the pretreated Ti6Al4V and deposition products (D30–D70).

Figure 2 shows XRD patterns of pretreated Ti6Al4V and D30–D70 obtained after deposition at different temperatures. It was found that a non-stoichiometric titanium oxide phase (Ti_6_O, JCPDS No. 73-1118) was produced in the pretreated Ti6Al4V due to the heat pretreatment of the substrate. It was expected to serve as a transitional material to enhance the adhesion between calcium phosphate and the alloy substrate because of its physicochemical similarities to them. This phase remained after deposition, irrespective of deposition temperature. By comparing the XRD patterns of D30–D70 with that of the pretreated titanium alloy, it was also identified that the Sr and F co-doped HA phase, i.e., Ca_9.37_Sr_0.63_(PO_4_)_6_F_2_ (JCPDS No. 79-7459) or SrFHA, was formed and coated on the pretreated Ti6Al4V, due to the following reaction during the deposition process [12]:

(10 − x) Ca^2+^ + 6 PO_4_^3−^ + x Sr^2+^ + y F^−^ + (2 − y) OH^−^ = Ca_(10−x)_Sr_x_ (PO_4_)_6_OH _(2−y)_ F_y_↓ (3)

The above new phase was not observed on D30 and D70. In other words, it was difficult to complete deposition at too low or high deposition temperatures. On the contrary, only moderate temperatures (40−60 °C) were suitable for the generation of SrFHA, as revealed by the XRD patterns of D40, D50, and D60.

**Figure 3:**
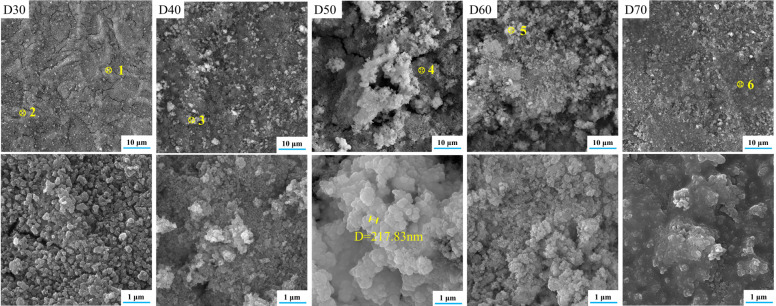
SEM images of the deposition products (D30–D70).

**Figure 4:**
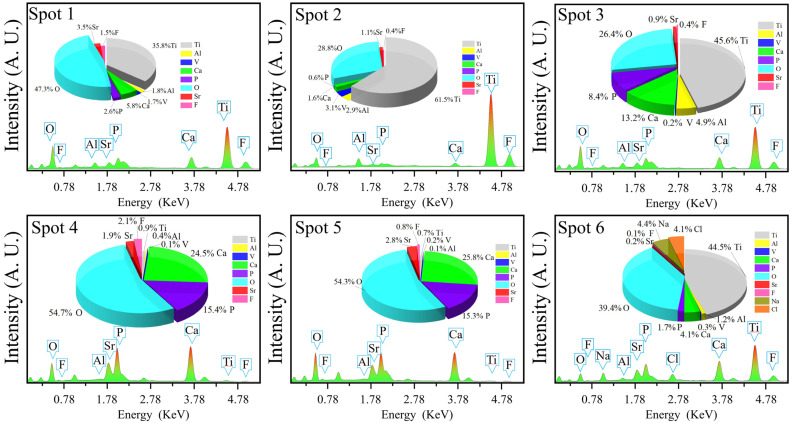
EDS analyses of the deposition products (D30–D70).

Figure 3 and Figure 4 show the microstructures and elemental distributions of D30–D70, respectively. For D30, there were no evident SrFHA particles deposited on the alloy substrate. Instead, the surface of D30 was mainly covered by titanium oxide particles about 300 nm in size, as confirmed by the EDS analysis of spots 1 and 2. When the deposition temperature increased from 30 °C to 40 °C, the deposition amount of SrFHA on the alloy gradually increased. It remained uniform but discontinuous. Note that the bright blocky particles on D40 were composed of newly generated SrFHA, as verified by the EDS analysis of spot 3. When the temperature increased further to 50 °C and 60 °C, massive SrFHA precipitated and aggregated to form a continuous coating. Moreover, the average particle size of SrFHA in D50 was larger than that in D60. When the deposition temperature continuously increased to 70 °C, there was heavy evaporation of the solution. Hence, the stable deposition of SrFHA on the Ti6Al4V substrate became difficult due to the intense liquid flow movement [9]. Consequently, the deposition amount of SrFHA sharply decreased. This observation was consistent with the XRD patterns in Figure 2. 

**Figure 5:**
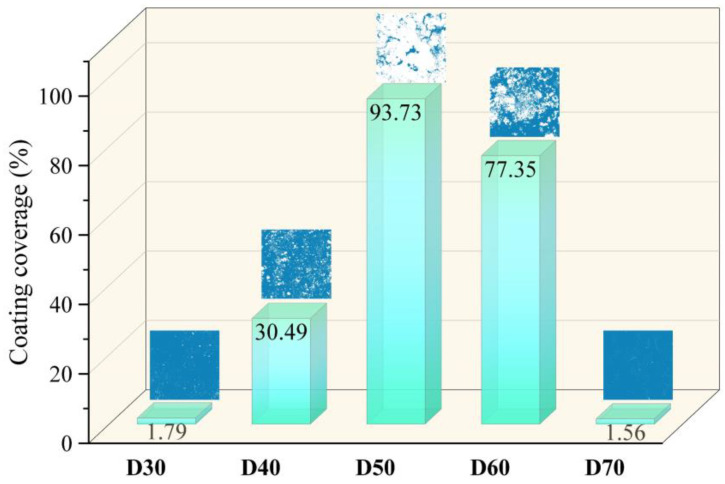
Coating coverages of the deposition products (D30–D70).

In order to assess the coating efficiency, the SrFHA coating coverage was determined by calculating the area ratio of the phase covered on the substrate after binarization of relevant SEM images. As shown in Figure 5, the coating coverage of the deposition products (D30–D70) initially increased and then declined with increasing temperature. In particular, the coating coverages of D30 and D70 were only 1.79% and 1.56%, while that of D50 reached the maximum value of 93.73%.

The above results show that the suitable deposition temperature was 50 °C because a more continuous hydroxyapatite coating was formed. It was speculated that properly increasing temperature under microwave irradiation could promote the formation of hydroxyapatite and the coating on the Ti6Al4V substrate which required a stable environment. In fact, microwave energy has been widely used for preparing composites of Ti6Al4V and pure HA or other calcium phosphates. Compared with conventional deposition, microwave radiation can promote the formation of more calcium phosphate nuclei from the solution mainly due to the selective microwave absorption of the phosphate which spontaneously crystallizes on the substrate surface [24]. This feature encourages the point-by-point deposition of calcium phosphate nuclei to build a crack-free coating on the pretreated Ti6Al4V surface as opposed to typical phosphate growth in the conventional process. Additionally, due to the high microwave dielectric loss factor of the solvent (water) [39], the deposition can be completed at a moderate temperature within a period as short as 5 min, in comparison with dozens of minutes or a few hours in conventional deposition studies [12].

**Figure 6:**
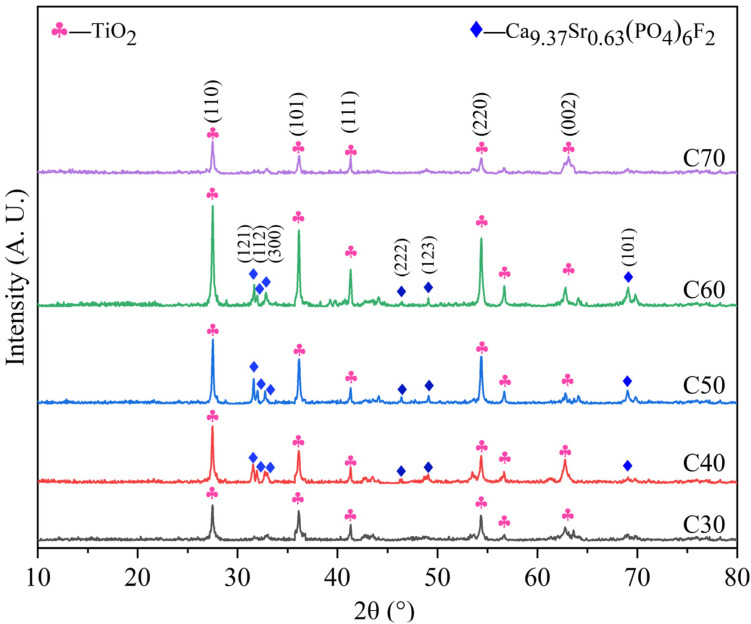
XRD patterns of the calcination products (C30–C70).

After calcination, the deposition products were expected to have phase and morphological changes. Figure 6 shows the phase compositions of C30–C70. The diffraction peaks of titanium oxide (TiO_2_, JCPDS No. 99–0090) were detected in all samples, indicating the conversion of nonstoichiometric titanium oxide to titanium oxide with high crystallinity after microwave-assisted calcination. The diffraction peaks of SrFHA were detected in C40, C50, and C60, which confirmed that the phase did not decompose after calcination. Instead, its crystallinity was improved, as demonstrated by peak narrowing in Figure 6. The lattice parameters of SrFHA were calculated to be a = b = 0.9413 nm and c = 0.6859 nm, indicating a shrinkage compared with the standard nano-HA crystal (a = b = 0.9418 nm and c = 0.6884 nm) [40]. The results were in accordance with the calculated lattice strain of 0.54%. These findings were believed to be caused by the dominant effect of fluorine ions which induced the contraction of the crystal and by the weaker impact of strontium ions which led to expansion of the crystal, due to its smaller doping amount [41].

**Figure 7:**
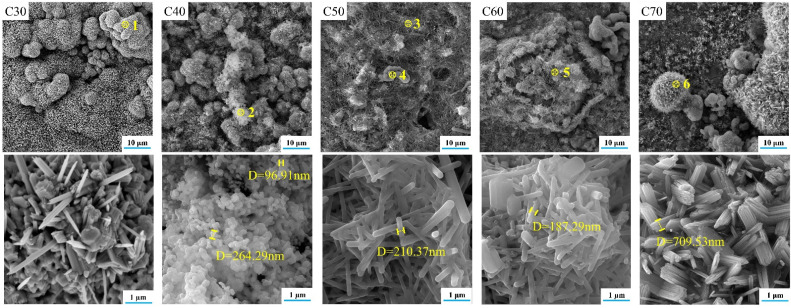
SEM images of the calcination products (C30–C70).

**Figure 8:**
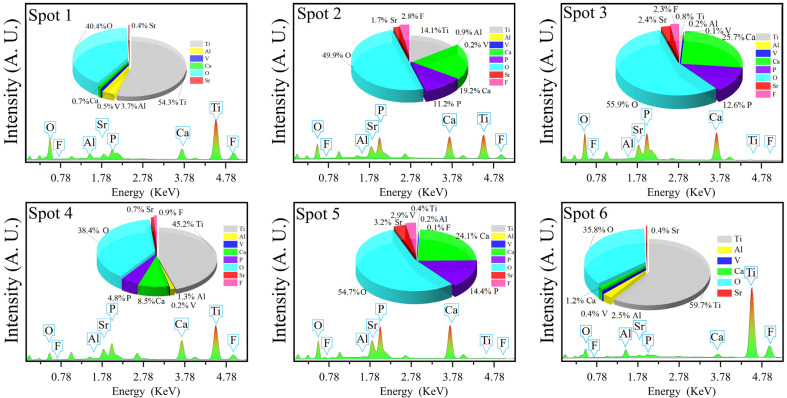
EDS analyses of the calcination products (C30–C70).

Along with the improvement of crystallinity, the material usually undergoes a perfection process of the crystal structure. In the present study, this process was partially revealed by the SEM images and EDS analysis of C30–C70, as shown in Figure 7 and Figure 8, respectively. For C30, there were needle-like titanium oxide particles with a diameter greater than 5 μm irregularly distributed on the substrate. Their generation was attributed to the evolution of nonstoichiometric titanium oxide in D30 during calcination. For C40, SrFHA whiskers were identified. Their formation was in accordance with the preferred orientation of the phase in the XRD pattern. For C50 and C60, large quantities of SrFHA were found and closely interwoven with titanium oxide on the substrate to form submicron whiskers with a diameter of about 200 nm after calcination. For C70, it showed similar micro-morphology to that of C30 due to the similarity of their phase compositions, as previously discussed. Overall, C50 showed the most suitable microstructure because of its largest coverage of SrFHA with submicron particle size. Rapid microwave-assisted calcination could improve the adhesion between the coating and substrate and crystallinity of SrFHA which would slow its dissolution after dental implantation. During the short processing time, excessive growth of the hydroxyapatite whiskers could also be prevented to ensure good bioactivity which was found to decline with the increasing grain size of HA [41].

**Figure 9:**
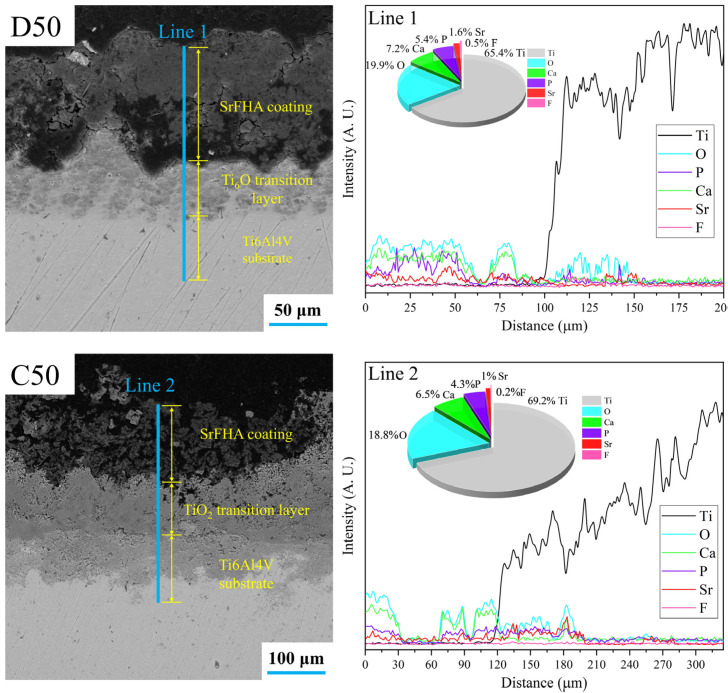
SEM images and EDS line scan analyses of cross-sections of coatings of the deposition product (D50) and calcination product (C50).

After deposition and calcination, the distributions and thicknesses of the SrFHA coatings were determined based on the SEM images and EDS line scan analyses of cross-sections of coatings of D50 and C50. The results are shown in Figure 9. The SrFHA coating with the thickness of nearly 100 μm was deposited on the titanium alloy substrate in D50, in addition to a slightly oxidized transition layer (Ti_6_O). After calcination, a more porous structure appeared in the coating, increasing the coating thickness to almost 130 μm which was much larger than those reported in the literature [42,43]. Moreover, an evident titanium oxide transition layer was identified. These observations agreed with corresponding EDS line scan analyses of cross-sections of D50 and C50 in Figure 9.

**Figure 10:**
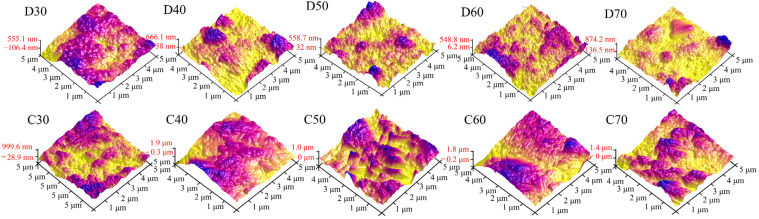
AFM images of the deposition products (D30–D70) and calcination products (C30–C70).

**Figure 11:**
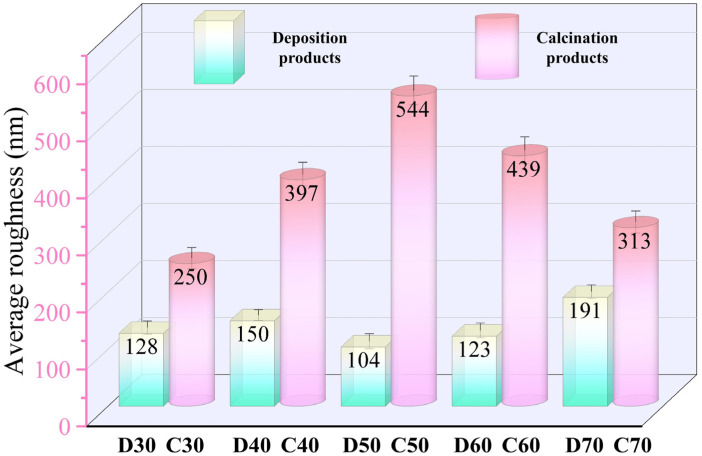
Average roughness of the deposition products (D30–D70) and calcination products (C30–C70).

To study the changes in surface morphology of the coatings in more detail, Figure 10 and Figure 11 show the AFM images and surface roughness of D30–D70 and C30–C70, respectively. For the deposition products D30−D60, they showed close values of average roughness. However, for D70, it possessed much higher roughness, probably associated with its unstable deposition process. Unlike the deposition products, the average surface roughness of C30–C70 initially increased and then declined as the deposition temperature increased. C50 showed the maximum average roughness of 544 nm. This was mainly because the generated SrFHA on C50 was transformed into whiskers with high crystallinity, which significantly increased the surface roughness. Note that the influence of the structural evolution of titanium oxide on the surface roughness was not as great as that of the SrFHA crystals due to its lower content. On the whole, the average roughness of the products after calcination was significantly enhanced, which could improve both mechanical properties, such as adhesion strength, and biological properties because enhanced surface roughness promotes the ability of cells to adhere and grow on the material [44].

### 3.2. Properties of Ti6Al4V/SrFHA Composites

#### 3.2.1. Adhesion Strength and Microhardness 

**Figure 12:**
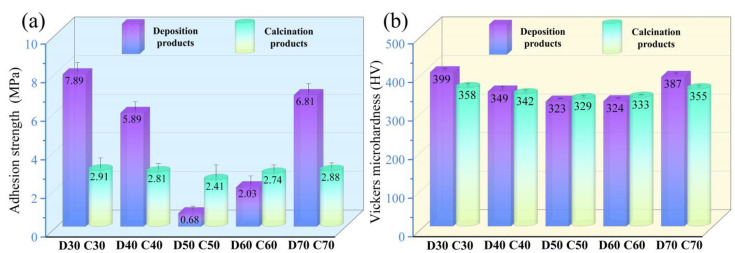
(**a**) Adhesion strength and (**b**) Vickers microhardness of the deposition products (D30–D70) and calcination products (C30–C70).

The adhesion strength and Vickers microhardness of D30–D70 and C30–C70 are shown in Figure 12. Evidently, D50 showed the lowest adhesion strength, 0.68 MPa, due to the highest amount of SrFHA. It should be mentioned that the higher adhesion strength of other deposition products was not an effective indicator of adhesion of the SrFHA coating to the substrate due to much less amount of deposited SrFHA. For instance, for D30 and D70, their adhesion strength was close to that of the titanium alloy to the binder instead of the coating. For the same reason, the Vickers microhardness of deposition products showed a similar changing trend. It varied within a limited range from 323 HV to 399 HV, all meeting the requirement for dental applications [45]. Some samples even showed a higher Vickers microhardness than Ti6Al4V (380.5 HV) [46]. For the calcination products (C30–C70), they showed smaller variations of adhesion strength and Vickers microhardness. Their adhesion strength varied between 2.41 MPa (C50) and 2.91 MPa (C30), all higher than (1.9 MPa) the similar composite previously reported [41]. C30, C40, and C70 possessed lower adhesion strength than the corresponding deposition products mainly due to the formation of titanium oxide. However, the other calcination products, especially C50, showed higher adhesion strength than the corresponding deposition products. It could be attributed to mechanical interlocking associated with the higher crystallinity of SrFHA and larger surface roughness and to chemical bonding due to the formation of titanium oxide whose whiskers, in turn, intertwined with SrFHA whiskers to further enhance mechanical interlocking. Eventually, there was a stronger adhesion of the coating to the substrate after calcination [34,47]. The change in Vickers microhardness of C30–C70 was basically consistent with that of D30–D70, in close association with the above changes of titanium oxide and SrFHA on the substrate. In particular, the Vickers microhardness of C50 was a little higher than that of D50 (329 HV vs. 323 HV).

#### 3.2.2. Wettability and In Vitro Apatite-Forming Capability

**Figure 13:**
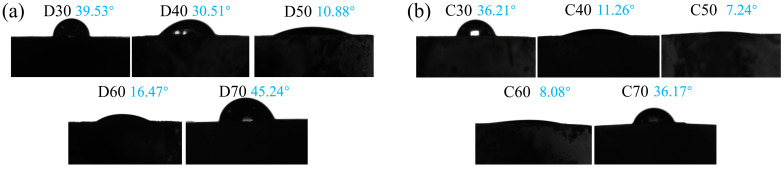
Water contact angles of (**a**) the deposition products (D30–D70) and (**b**) the calcination products (C30–C70).

The biological activity of a material can be revealed by its wettability and in vitro apatite-forming capability [45,48]. For determination of wettability, the water contact angles of all deposition and calcination products were measured. As shown in Figure 13, the contact angles of all deposition products (D30–D70) were lower than 50°, with the lowest value 10.17° achieved when the deposition temperature was 50 °C. In fact, the water contact angle of D50 was even lower than that of pure HA synthesized under the same conditions (11.70°) and in the previous study [43], indicating the positive influence of doping strontium and fluorine ions into HA. Evidently, this result also agreed with the initial increase and then decrease of coverage of the SrFHA coating on the Ti6Al4V substrate. The water contact angles of the calcination products (C30–C70) were lower than those of corresponding deposition products, with the same changing trend. Among them, C50 showed the smallest water contact angle (7.24°). The results showed that more SrFHA improved the hydrophilicity of the composite.

**Figure 14:**
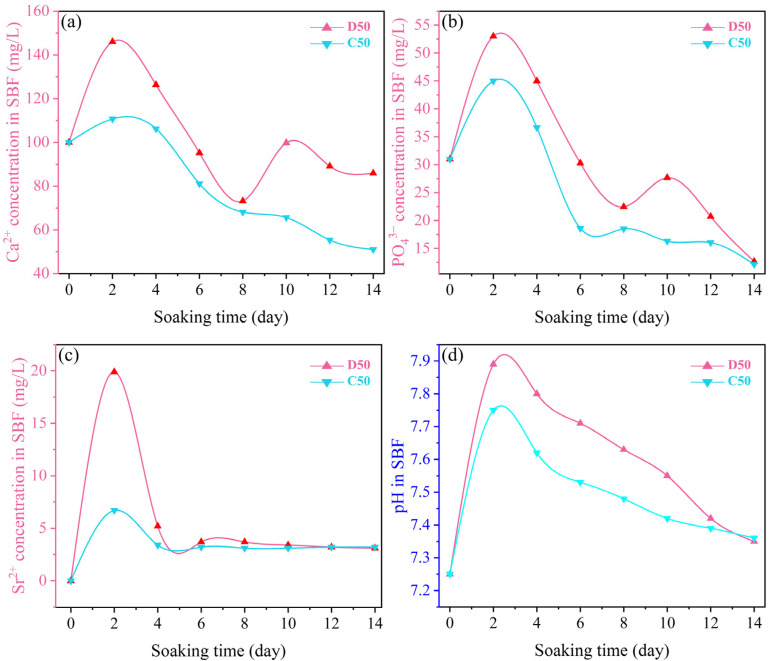
Variations of (**a**) Ca^2+^, (**b**) PO_4_^3−^ and (**c**) Sr^2+^ concentrations, and (**d**) pH in the SBF after immersion of the deposition product (D50) and calcination product (C50).

For characterizing the in vitro apatite-forming capabilities of the products, the changes in ion concentrations in the SBF after the immersion of D50 and C50 were measured. The results are shown in Figure 14. In the SBF immersing D50, the concentrations of Ca^2+^ and PO_4_^3−^ significantly increased on day 2 due to the partial dissolution and release from the coating. It decreased within 2–8 days, as a result of inducing new HA. It was followed by fluctuations in 8–14 days, indicating the repeated dissolution and formation of HA. In the SBF immersing C50, the concentrations of Ca^2+^ and PO_4_^3−^ increased at the initial stage of immersion (day 2), but the range was smaller than that of D50. It continued to decline between day 2 and day 14, without repetitive dissolution and precipitation. This was because the hydroxyapatite in the calcination product showed higher crystallinity, which restrained dissolution. It was also found that D50 and C50 showed the same changing trend of Sr^2+^ concentration in the SBF. They dissolved in the first 2 days and then formed precipitates until day 14. Due to its low contents in D50 and C50, the concentration of Sr^2+^ fluctuated at a low level (about 3.1 mg/mL). It is also worth noting that the concentration of F^−^ was not monitored in this study due to the inability of ICP-OES for the measurement. 

Figure 14 also shows the variations of pH of SBF after the immersion of D50 and C50 for different periods of time. For D50, the pH value rose to the maximum of 7.8 and 7.9, respectively, on day 2 and continued to decline from day 4 to day 14, indicating that Ca^2+^ was firstly dissolved and released from the coating into the SBF. Due to the exchange between Ca^2+^ and H^+^, the OH^−^ concentration increased, giving rise to a higher pH value. When the concentration of Ca^2+^ in the SBF reached the maximum, the pH value was the maximum. The apatite spontaneously formed on the coating surface. Finally, the concentrations of Ca^2+^ and PO_4_^3−^ in the SBF decreased, producing a bone-like layer that reduced the pH value [32].

**Figure 15:**
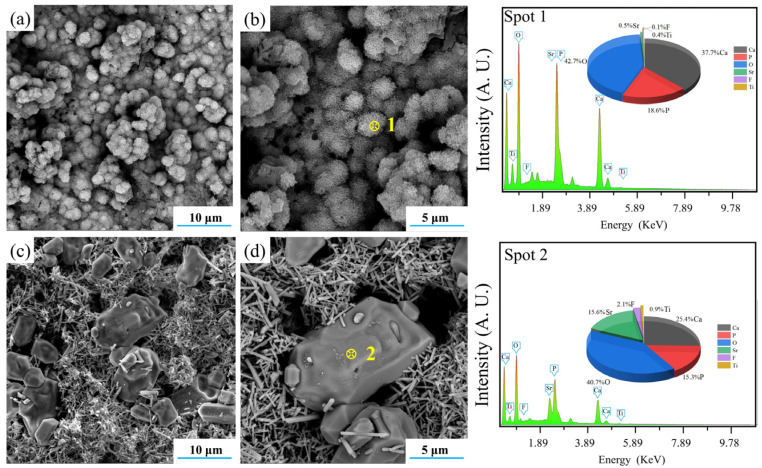
SEM–EDS analyses of (**a**,**b**) the deposition product (D50) and (**c**,**d**) the calcination product (C50).

The ability of D50 and C50 to induce the formation of hydroxyapatite is also clearly shown in Figure 15. It was obvious that both of them could induce the formation of HA with different morphologies. After immersion in the SBF for 14 days, D50 induced the formation of HA flakes which assembled into larger spherical clusters, while C50 induced the formation of more and larger blocky HA particles.

The above analysis showed that the Ti6Al4V/SrFHA composite could be rapidly synthesized at a moderate temperature by microwave-assisted liquid phase deposition. The subsequent calcination elevated the crystallinity of SrFHA and surface roughness, which increased the adhesion strength and microhardness of the composite. Doping strontium and fluorine ions into HA to produce an SrFHA coating on the alloy substrate enhanced the wettability and in vitro apatite-forming capability of the composite. 

## 4. Conclusions

In this study, the feasibility of synthesis of Ti6Al4V/SrFHA composites via coating strontium and fluorine co-doped HA onto the Ti6Al4V substrate by microwave-assisted liquid phase deposition and calcination was examined. Particular attention was paid to the effects of the deposition temperature on the preparation process and product properties. It was found that the SrFHA coating was not obtained at the deposition temperatures of 30 °C and 70 °C. Instead, the coating was only available when the deposition was carried out at 40−60 °C. At the optimal deposition temperature of 50 °C, the coating with the largest uniform continuous SrFHA coverage was obtained. After calcination of the deposition product, the adhesion strength and Vickers microhardness of the Ti6Al4V/SrFHA composite increased from 0.68 MPa and 323 HV to 2.41 MPa and 329 HV, respectively. It was superior to the counterpart previously reported and met the requirement of dental applications. Meanwhile, there was a decrease of water contact angle from 10.88° to 7.24°, showing the excellent wettability of the composite. Moreover, based on the SBF test, the composite showed good biological activity. More detailed exploration of the biological properties of the composite is ongoing. The great performance of the composite obtained at the deposition temperature of 50 °C was mainly a result of the combined effect of the co-doping of strontium and fluorine, high crystallinity of SrFHA, large surface roughness, and formation of the titanium oxide transition layer. The results provide a useful guide for the synthesis of novel dental implant materials.

## Data Availability

The data presented in this study are available on request from the corresponding author.
